# Potential Activity of *Arthrospira platensis* as Antioxidant, Cytotoxic and Antifungal against Some Skin Diseases: Topical Cream Application

**DOI:** 10.3390/md21030160

**Published:** 2023-02-27

**Authors:** Saly Gheda, Eman H. F. Abd El-Zaher, Alaa M. Abou-Zeid, Nesma A. Bedair, Leonel Pereira

**Affiliations:** 1 Botany Department, Faculty of Science, Tanta University, Tanta 31527, Egypt; 2 MARE—Marine and Environmental Sciences Centre, ARNET, Department of Life Sciences, University of Coimbra, Calçada Martim de Freitas, 3000-456 Coimbra, Portugal

**Keywords:** cyanobacteria, antifungal, dermatophyte, antioxidant, cytotoxicity

## Abstract

This research evaluated the antifungal effectiveness of *Arthrospira platensis* ethanol, methanol, ethyl acetate and acetone extracts against the tested pathogenic fungi (*Candida albicans*, *Trichophyton rubrum* and *Malassezia furfur*). Antioxidant and cytotoxicity effectiveness of *A. platensis* extracts against four distinct cell lines were also assessed. Methanol extract of *A. platensis* exhibited the highest inhibition zones against *Candida albicans* as measured by the well diffusion method. A transmission electron micrograph of the treated group of *Candida* cells with *A. platensis* methanolic extract showed mild lysis and vacuolation of the cytoplasmic organelles. In vivo, after induced infection of mice by *C. albicans* and treatment with *A. platensis* methanolic extract cream, the skin layer emerged with the removal of *Candida* spherical plastopores. The extract of *A. platensis* recorded the highest antioxidant activity using the DPPH (2, 2- diphenyl-1-picrylhydrazyl) scavenging method (IC_50_ 28 mg/mL). A cytotoxicity test using a MTT assay showed that the *A. platensis* extract had strong cytotoxic activity against the HepG2 cell line (IC_50_ 20.56 ± 1.7 μg/mL) and moderate cytotoxic activity against MCF7 and the Hela cell (IC_50_ 27.99 ± 2.1 μg/mL). Gas Chromatography/Mass Spectroscopy (GC/MS) results revealed that the effective activity of *A. platensis* extract could be linked to a synergistic impact between their prominent composition as alkaloids, phytol, fatty acids hydrocarbons, phenolics and phthalates. *A. platensis* extract contains active metabolites that constitute a promising source of antifungal, antioxidant and anti-proliferative compounds for the pharmaceutical drug industry.

## 1. Introduction

Dermatophytes are a kind of filamentous fungi that may enter keratinized tissues in humans and other animals, causing superficial sores. *Trichophyton rubrum* and *Malassezia furfur* are the most common dermatophyte species in fungal infections, generating a variety of clinical conditions across the world [1]. Yeasts, which are considered opportunistic agents, especially *Candida* species, are another major category of pathogenic fungus that is usually detected in onychomycosis, vulvovaginitis and other mucus–cutaneous frameworks [2]. However, invasive fungal infections caused by species of *Candida* are a growing clinical issue [3]. Infections with pathogenic fungi have grown during the last two decades. Though bacteria are responsible for the majority of infectious episodes, fungi, especially *Candida albicans*, produce more than 80% of all fungal infections and account for more than 20% of fatal infections in patients with leukemia and 13% in patients with lymphoma. The bulk of human fungal infections are caused by *Candida albicans*, an important aerobic eukaryotic pathogen. These infections range from systemic infections that can be fatal in people with weakened immune systems, such as cancer patients, to candidiasis in immunocompetent colonized hosts [4].

One of the most serious global challenges is antimicrobial resistance (AMR). A threat to our capability to treat common infections is the evolution and spread of antimicrobial-resistant organisms and resistance mechanisms worldwide. A large rise in the number of infections is becoming increasingly challenging to treat due to treatment ineffectiveness [5]. For the treatment of fungi, the search for newer medications with natural ingredients has become more active because of the availability of only a few antifungal classes and the recent evolution of resistant strains. Synthetic substances must now give way to naturally produced active compounds. Several natural components are used, and various studies have confirmed the existence of bioactive components in algal extracts, specifically those effective at inhibiting fungal activity [6].

Proteins, carbohydrates, fats, minerals and polyunsaturated fatty acids, and biologically active substances such as carotenoids (carotene, xanthophylls), chlorophylls and phycobilins (phycocyanin and phycoerythrin), which have antioxidant, antiviral, antifungal, antibacterial, anti-inflammatory, antidiabetic and antitumor properties, are found in algae and their extracts [7,8]. While they reduce activity, these chemicals are harmless and do not leave deposits. This indicates that there is a need to create novel and safe biological products with characteristics similar to synthetics, especially antifungal, antibacterial, antioxidant substances and colorants [9]. 

A significant appeal of cyanobacteria, also known as blue–green microalgae, is that they are a natural source of bioactive substances with a variety of significant biomedical activities. *Arthrospira platensis* (formerly *Spirulina platensis*) is a cyanobacteria that is distinguished by the presence of several pigments including chlorophylls, phycobilins and carotenes. Unique photosynthetic pigments known as phycobiliproteins are plentiful in *Arthrospira*. In addition to their role as an antibacterial, antifungal, antiviral, antioxidant, anticancer and anti-inflammatory component, phycobiliproteins isolated from *Arthrospira* showed positive usage for enhancing the immune system and preventing development of cancer cells [10]. Several studies have suggested screening microalgae extracts to produce new antifungal components to treat infectious diseases as a potential replacement for or addition to chemical fungicides [11].

Antioxidants are substances that prevent or delay the oxidation process by scavenging or neutralizing free radicals in body cells [12]. They now play a significant role in the prevention and treatment of diseases linked to oxidative stress, such as diabetes, cancer and cardiovascular conditions [13]. To lessen the negative effects of free radicals, various synthetic antioxidants are employed in commercial products. However, these artificial antioxidants maybe have negative side effects [14]. The quest to find natural antioxidants to replace these synthetic ones has emerged as a crucial advance in immunity pharmacy research.

Various extracts of cyanobacterial strains having anticancer activity against the colon CT-26 and lung 3LL cell lines have been reported [15]. *A. platensis* dietary supplementation proved beneficial for both preventing and treating pancreatic cancer [16]. Several substances having effective mechanisms against a variety of cancer cell lines were derived from various algal and cyanobacterial species [17].

Studies demonstrating the role of *Arthrospira/Spirulina* as an antifungal for *Trichophyton rubrum* and *Malassezia furfur* are lacking. Most studies have clarified the role of *Arthrospira/Spirulina* as an antifungal only in vitro but not in vivo. Therefore, the goal of this research is to determine how cyanobacteria *Arthrospira platensis* extracts affect the growth of some dermatophytes (*Candida albicans*, *Trichophyton rubrum* and *Malassezia furfur*) in vitro and in vivo in order to show the effectiveness and safety of *A. platensis* methanolic extract cream when applied topically to mouse skin. The antioxidant and cytotoxic properties of cyanobacterial extract were also evaluated.

## 2. Results

### 2.1. Cultivation and Growth Curve of Arthrospira platensis 

**Figure 1:**
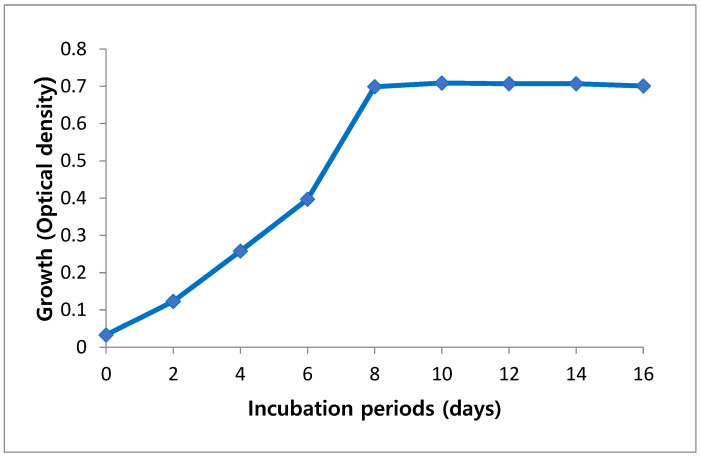
Growth curve of *Arthrospira platensis* cultivated on Zarrouk medium during incubation period.

Growth of *A. platensis* was estimated as an optical density (OD) to detect the best period needed for their maximum growth. Growth analysis of cultures was monitored every two days. Maximum optical density that indicates a maximum biomass concentration of 0.720 was observed at day 10, but further reduction in growth rate was observed at day 12 (Figure 1). 

### 2.2. In Vitro Study

#### Antifungal Activity of the Cyanobacterial Extracts

Cyanobacterial extracts having antifungal properties against the fungal species examined are presented in Table 1 which shows that methanol was the best solvent. The highest inhibition zones were detected with *Arthrospira platensis* methanolic extract against *Candida albicans* (19.2 mm) followed by *Malassezia furfur* (17.3 mm) and then *Trichophyton rubrum* (11 mm).

On the other hand, ethyl acetate strongly affected *C. albicans* with an inhibition zone of 16 mm, then *M. furfur* with an inhibition zone of 12.6 mm and did not affect *T. rubrum*; meanwhile acetone recorded an inhibition zone of 16.3 mm with *C. albicans*, 10.7 mm with M. furfur and showed no effects with *T. rubrum*.

Finally, ethanol affected *M. furfur* followed by *C. albicans* then *T. rubrum* with inhibition zones of 12, 11.3 and 10 mm, respectively. *Arthrospira platensis* extracts with different solvents affected the growth of the tested fungi and displayed different inhibition zones, even in the case of ethanol, acetone and ethyl acetate solvents. We used 70% of different solvents (methanol, ethanol, acetone and ethyl-acetate) as a negative control The best solvent was methanol and the best fungus affected by the extracts was the *Candida* fungus; both were chosen for the in vivo study. Molecular identification of *Candida* proved it *Candida albicans* AUMC No. 13531.

### 2.3. Minimal Inhibitory Concentration (MIC) Assay

**Figure 2:**
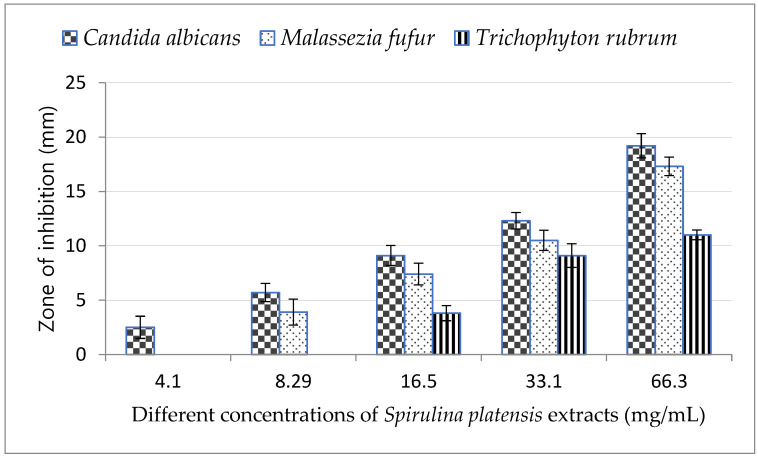
Minimum inhibitory concentration (MIC) of *Arthrospira platensis* extracts on tested fungi.

The lowest concentration of *Arthrospira platensis* methanolic extract (4.1 mg/mL) inhibited only the growth of *C. albicans* and was not effective against *M. furfur* and *T. rubrum* as shown in Figure 2, while 8.29 mg/mL of *A. platensis* methanolic extract was effective against both *C. albicans* and *M. furfur*. 

By increasing the concentration of *A. platensis* methanolic extract, the growth inhibition of all tested fungi increased and reached a maximum at concentration of 66 mg/mL; the highest inhibition zone was observed in *C. albicans*.

### 2.4. Treated Candida albicans under Transmission Electron Microscopy (TEM)

**Figure 3:**
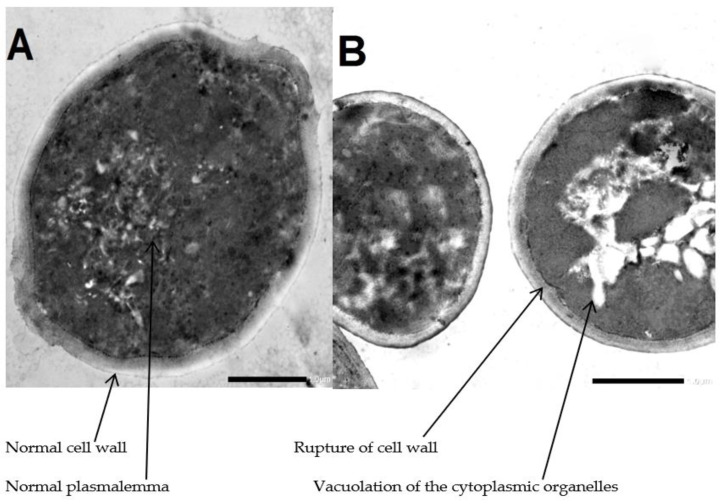
Transmission electron micrograph. (**A**) Untreated *Candida* conidia. (**B**) Treated *Candida* conidia by *Arthrospira platensis* extracts. Mag (×40,000).

As shown in Figure 3, the transmission electron micrograph of the control group of *Candida* conidia showed the normal ultrastructure of *C. albicans* including normal cell wall, plasmalemma and homogeneous cytoplasm. Meanwhile, the transmission electron micrograph of treated group of *Candida* conidia with *A. platensis* methanolic extract showed mild lysis and vacuolation of the cytoplasmic organelles and rupture of the cell wall.

### 2.5. In Vivo Study

#### Effect of *Arthrospira platensis* Extract on the Treatment of Wounded Mice Artificially Infected with *Candida albicans*

**Figure 4:**
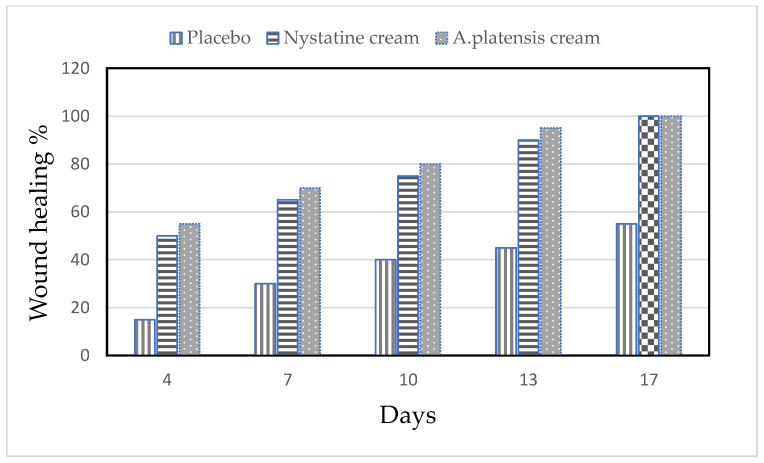
Efficacy of wound healing in mice infected with *Candida albicans* using different creams.

**Figure 5:**
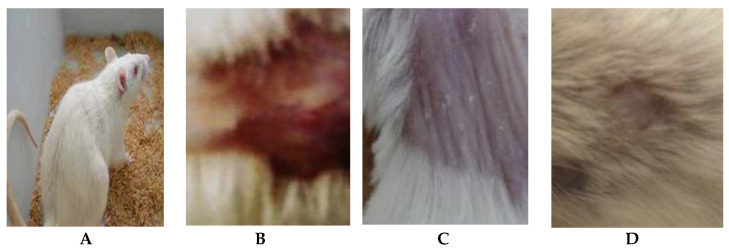
Efficacy assessment of wound healing in mice infected with *C. albicans* after 17 days of infection. (**A**) Healthy mice, (**B**) Placebo treatment, (**C**) Nystatin treatment and (**D**) *Arthrospira platensis* extract cream.

The alleviation of the wound area in the placebo treated group was low when compared to the *A. platensis* extract cream- and nystatin-treated groups (G3, G4). The healing of the wound recorded for nystatin was 50–90% from days 4 to 13 after treatment. However, *A. platensis* extract cream caused wound healing of 55–95% from days 4 to 13 after treatment, as shown in Table 2 and Figure 4 and Figure 5. The data detected that the maximal healing of wounds was by *A. platensis* cream as compared with nystatin and the placebo cream base. The lowest redness of wound and moderate hair growth was observed after 13 days of applying *A. platensis* extract cream; moreover, at the end of the 17-day study period, there was complete removal of inflammation and massive hair growth.

### 2.6. Histopathological Examination of Mouse Skin Tissues

**Figure 6:**
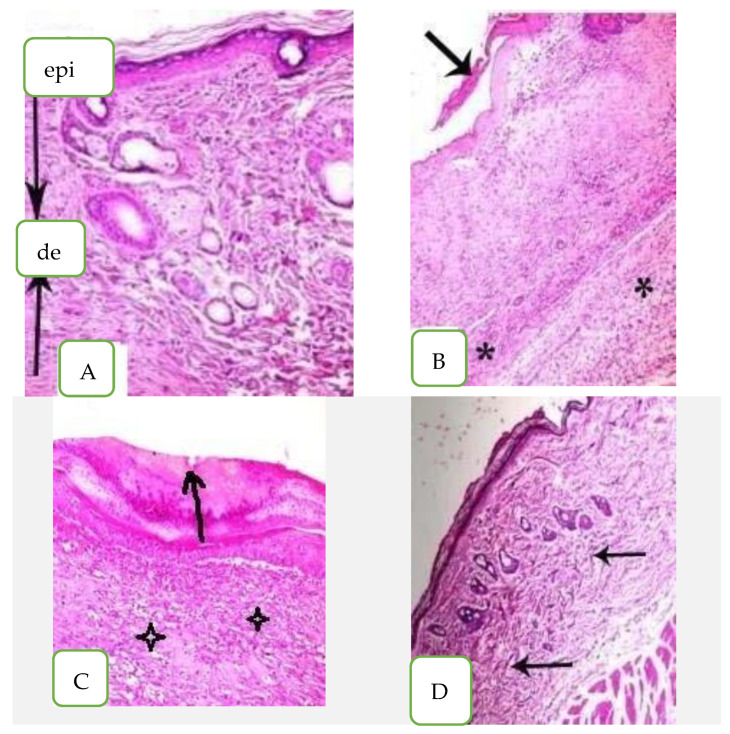
Histopathological examination of skin mice groups. (**A**) Healthy mice, (epi) epidermis layer, (de) dermis layer, (**B**) Infected non-treated skin, (**C**) Nystatin treatment and (**D**) *Arthrospira platensis* extract cream.

In the histopathological analysis of healthy mouse skin tissues, the epidermis (Epi) looked normal with keratinized stratum corneum. The dermis layer (D) appeared with fibers consistently spaced and condensed without any disruption as shown in Figure 6A. On the other hand, the histological section of mice infected with *C. albicans* (untreated) looked incomplete, with several layers of epidermis (arrow) and rounded, short, elongated cells; there was some hypha swelling and keratinized fibers that were loose and disordered in the stratum corneum. The epidermis also had a chronic inflammatory cellular infiltration made up primarily of lymphocytes and plasma cells (star) (Figure 6B). The nystatin-treated skin sections possessed abnormal epidermis as the keratinized fibers of stratum corneum were still disrupted and had separation of the epidermis (arrow). The dermis also showed edema with some inflammatory cellular infiltrate of lymphocytes (star) as seen in Figure 6C.

The skin section of *A. platensis* extract cream exhibited no hypha swelling and no significant toxic effects when compared to the control. The skin tissue appeared normal with normal epidermis and keratinized fibers of stratum conium which were regularly arranged; they also appeared condensed without any disruption and the dermis appeared normal with minimal inflammatory cellular infiltration (arrow) as shown in Figure 6D.

### 2.7. Antioxidant Potential of Arthrospira platensis Methanolic Extract

#### DPPH Radical Scavenging Activity

The DPPH technique was used to test the *Arthrospira platensis* methanolic extract antioxidant activity, and the results were expressed as a percentage and an IC_50_, i.e., the amount of extract needed to scavenge 50% of the DPPH radical. The lowest IC_50_ identifies the strongest level of antioxidant activity. Results in Table 3 recorded the highest scavenging activity of the DPPH radical with *A. platensis* methanolic extract and an IC_50_ of 28 µg/mL.

In the current investigation, varied doses of crude extract demonstrated relatively equal DPPH antioxidant activity when compared to ascorbic acid as the reference antioxidant, with an IC_50_ value of 22 g/mL. *A. platensis* methanolic extract had the greatest DPPH radical scavenging activity with 79.7%. It was found that increasing the extract concentration increased the DPPH radical’s capacity to scavenge oxygen.

### 2.8. Cytotoxicity Assay

**Figure 7:**
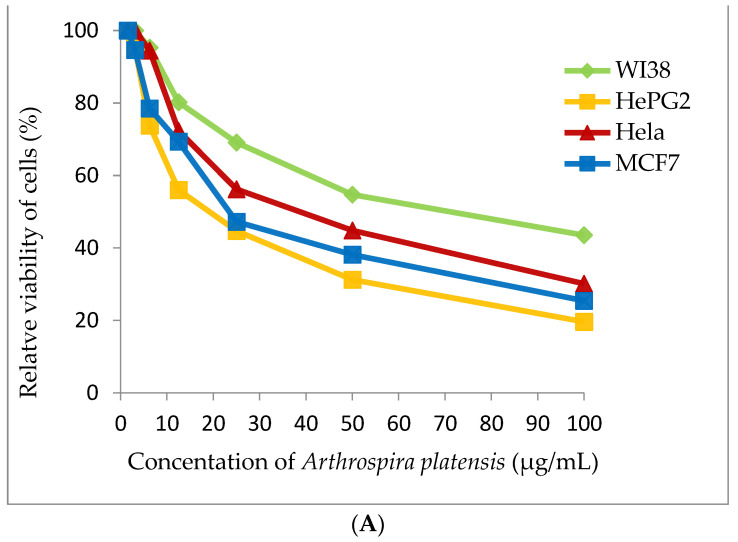

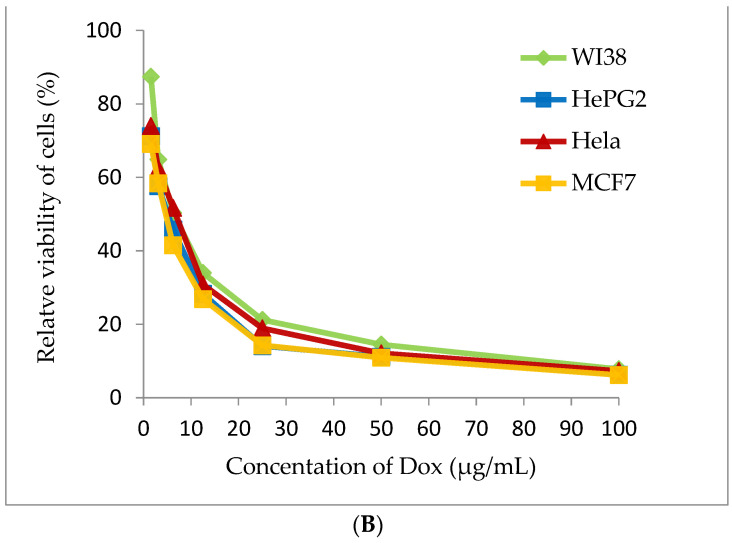
Cytotoxicity assay (**A**) *Arthrospira platensis* methanolic extract, (**B**) Doxorubicin (standard anticancer) on different cell lines by MTT method.

Cyanobacterial extract was selected for cytotoxic assay for four tested human tumor cell lines (WI38, HePG2, Hela and MCF7). Methanolic extract of *A. platensis* demonstrates strong cytotoxic activity against the HePG2 cell line (IC_50_ 20.56 ± 1.7 μg/mL) and moderate cytotoxic activity against MCF7 and the Hela cell (IC_50_ 27.99 ± 2.1 and 38.91 ± 2.4 μg/mL), respectively. *A. platensis* extract showed weak cytotoxic activity against the WI38 cell line (IC_50_ 65.82 ± 3.5 μg/mL) (Table 4 and Figure 7).

### 2.9. GC-MS Analysis of Arthrospira platensis Methanolic Extract

Methanolic extract of *A. platensis* as presented in Table 5 included various important biomolecules of antifungal, antioxidant and antitumor potent activity. The GC-MS analysis of *A. platensis* methanolic extract detected that the active components were heptadecane (100.00%), 3,7,11,15-tetramethyl-2-hexadecen-1-ol (14.34%), triacetin (2.34%), (methyl palmitate) hexadecanoic acid, methyl ester (1.54%), 4-hydroxy-4-methyl-4H-naphthalen-1-one (1.46%), (dihydroactinidiolide) 2(4H)-benzofuranone, 5,6,7,7a-tetrahydro-4,4,7atrimethyl-, (1.33%).

## 3. Discussion

Cyanobacteria are rich in many bioactive compounds and have become a cheap and safe source of these compounds in many medicinal and pharmacological applications [18]. However, some variables, such as environment, season of collection and culture, growth stages and experimental procedures may impact cyanobacteria’s antifungal activity [19]. Although many studies have used several solvents to screen cyanobacteria for antimicrobial activity, it is still unknown which solvent is the most successful and acceptable for extracting algae for antifungal applications [10].

The methanol extract of *Arthrospira platensis* used in the current investigation was the most significant solvent when compared with the other applied extracts; it had the strongest antimicrobial activity against all tested fungal species using the well diffusion method. It was also observed that methanol extract showed the highest inhibitory activity, perhaps due to its high polarity; it also allows the extraction of many specific compounds. These results indicated that the antimicrobial activity of the extracts depended mainly on the type of cyanobacterial species, the solvent used and the tested pathogen. These outcomes aligned with those of Musbah et al. [20] who confirmed that methanol and ethanol extracts are more effective than other solvents used in extracting antimicrobial compounds from *A. platensis*. [21] Usharani et al. estimated that the methanol extract of *A. platensis* displays the strongest antifungal effect against some fungal species such as *Candida glabrata*, *Candida albicans*, *Candida tropicalis*, *Aspergillus fumigatus*, *Aspergillus niger* and *Aspergillus flavus*.

In contrast to the significant effects of the methanol and ethanol extracts of *A. platensis*, many researchers reported other extracts that are more effective against microbes. Gouda et al. [22] reported that *A. platensis* butanol extract is most effective than other tested solvents. Al-Saif et al. [23] found that chloroform was the most effective solvent. On the other hand, obtained data revealed that methanol was the most effective extraction solvent with significant antifungal activity.

In the present study, all fungal candidates were inhibited by *A. platensis* methanol extract; the highest inhibitory effect recorded against *C. albicans* had an inhibition zone of 19.2 mm and minimum inhibitory concentration of 8 mg/mL. These results agree with El Shouny et al. [24] who reported that methanol extract obtained from *A. platensis* showed antimicrobial activity. 

This was a similar finding to that of Abedin and Taha [25] who found that *A. platensis* methanol extract inhibited the growth of *Aspergillus niger*, *Aspergillus fumigatus* and *Candida kefyr*. 

*A. platensis* methanolic extract inhibited the growth of *C albicans*, *M. furfur* and *T. rubrum* at concentrations of 4, 8 and 16 mg/mL, respectively. Results were confirmed by Ibraheem et al. [26] who reported that *A. platensis* methanol extract was effective in reducing fungal species in a range of concentrations from 2 to 16 mg/mL.

The bioactive compounds present in cyanobacteria work against microbes through a variety of methods. As a type of natural phenol, flavonoids have the potential to interact with soluble and extracellular proteins in microbial cells. By weakening the membranes of microorganisms, terpenoids can also trigger the disintegration of their cell walls. Proteins and enzymes can leak from the cells as a result of saponins. Alkaloids inhibit the synthesis of nucleic acids and induce the modification of microbial cell membrane permeability; they also bring about the loss of internal macromolecules and cellular integrity, which eventually lead to final cell death [7].

*A. platensis* extract exhibits anticandidal activity, as demonstrated by electron microscope observations, but more research on the method of action of the extract on *C. albicans* spore cells is required before recommending its usage as a safe and efficient antifungal medication. The cell membrane of *C. albicans* treated with *A. platensis* methanolic extract remained intact, based on what was observed in an electron microscope. This demonstrates that *A. platensis* methanolic extract showed mild lysis and vacuolation of the cytoplasmic organelles; it also damages the fungal cell wall in the body while leaving the cell membrane undamaged. Currently, azoles, which target the membranes of the fungal cells and have a high level of toxicity and side effects as well as a high risk of developing drug resistance, are the therapeutic drugs most frequently used to treat *C. albicans* infections. TEM and SEM have been used in earlier studies to examine the morphological alterations brought on by treating *C. albicans* with various antifungal medications [27,28].

One of the typical fungal infections that affects organs is candidiasis. Lesions are caused by the yeast *C. albicans*, which is a member of the normal microflora and is carried by 30 to 50% of people [29]. In this work, mice were injected with a suspension of *C. albicans*, which caused macroscopic lesions and microscopic changes in the connective tissue and the epithelium underneath the inoculation region. These findings corroborated those of Abo Baker [30], who discovered that the injection of rats with *C. albicans* led to lesions in the infected area. In the current investigation, we employed *A. plantensis* because natural compounds offer a viable alternative to traditional antifungal medications, and the topical cream produced from methanolic extract of *A. platensis* accelerated wound healing and hair growth in mice. These results were in accordance with Marangoni et al. [31], who reported that *A. platensis* extract interacts with all strains of *Candida* and caused complete disappearance of *Candida* yeast; the beginning of hair regrowth and its return to normal appearance was observed after the mice started receiving *A. platensis* daily. Zamani et al. [32] investigated the efficacy of cream produced from purified *A. platensis* phycocyanin on mice infected with *C. albicans*. The accelerated healing effect was observed in infected mice treated with phycocyanin cream in comparison with the control group.

El-Sheekh et al. [33] also reported that *Arthrospira* contains a wide range of biological activities, including antifungal, antibacterial and antiviral properties.

As the usage of synthetic antioxidants is being questioned, there is an increasing interest in discovering alternative antioxidant agents; antioxidants derived from natural sources appear promising. Natural antioxidants discovered in cyanobacteria and microalgae and their preparations are of great interest [8]. When fresh, *A. platensis* contains effective antioxidant compounds, such as as glutathione and ascorbate (GSH), and secondary metabolites such as carotenoids, eckol and tocopherols. Furthermore, microalgae have been discovered to have antioxidant activity due to their high concentration of fatty acids, carotenoids and phenolic compounds [5].

Given their capacity to scavenge the DPPH radical, several algal extracts with various solvents were examined for their antioxidant potential. Depending on the species and solvent, *A. platensis* extracts were capable of converting the steady diphenyl picryl hydrazine radical to yellow DPPH. *A. platensis* methanol extract had the highest DPPH scavenging activity at 79.7 ± 0.21. The IC_50_ value for the methanol extract of *A. platensis* was 28 mg/mL. This result was consistent with what Scaglioni et al. [34] observed on the effects of *A. platensis* extracts which showed strong antioxidant activity.

Algal phytochemicals work in concert to potentially act as antioxidants by scavenging singlet oxygen, superoxide and hydroxyl radicals, and by binding to metal ions, donating electrons or hydrogen and stabilizing lipid peroxidation.

As a defense mechanism, cyanobacteria are renowned for their capacity to withstand oxidative stressors by activating both enzymatic and non-enzymatic antioxidants. These antioxidant compounds already had a well-established role in chemoprevention and tumor development regulation [13,16].

The present work investigated the cell survival of cancer cell lines using methanolic *Arthrospira* extract. The methanolic extracts of *A. platensis* showed varying degrees of inhibitory efficacy against the four evaluated human tumor cell lines. This agreed with Mashjoor et al. [35], Hernandez et al. [36] and Deviyani et al. [37] who reported cytotoxic potential and activity of the *A. platensis* extracts against three cell lines, including MCF7, HeLa and HepG2.

There is significant debate about the mechanism(s) through which *A. platensis* methanolic extracts cause tumor cell death. According to Dai et al. [38], fatty acids have the ability to kill tumors through a variety of mechanisms: (a) Increased ROS production; (b) Activation of caspase enzymes; (c) Accumulation of toxic byproducts of lipid peroxidation leading to cell apoptosis; (d) Activation of peroxisome proliferator-activated receptors (PPARs); (e) Altering the expression of genes/anti-oncogene; (f) Stimulation of cancer cells by chromos. Recently, Ahmed et al. [39] found that *A. platensis* methanolic extract significantly reduced the growth of hepatocellular carcinoma cells, stimulated apoptosis and arrested the cell cycle at various stages. Additionally, Ahmed et al. [40] found that *A. platensis* crude extract significantly affects breast cancer (HCT-116), lung cancer (A549), liver cancer (HepG-2) and colon cancer (HCT-116) (MCF-7).

The GC–MS profiles indicated that *A. platensis* methanolic extract was a consistent source of bioactive chemicals, and mass spectrometry (GC–MS) demonstrated the existence of a compound with antifungal, antioxidant, antitumor and anticancer properties [41]. The component proportions differed across various species. However, basic chemicals including fatty acids, alcohols such as phytol, phthalate and hydrocarbons were prevalent and might be responsible for the observed biological activity of these species. Several studies are in accordance with the studied work. 

Some discovered chemicals present in microalgae have biological activities, such as antifungal and antioxidant activities [42]. *A. platensis* extracts including phenolics, amino acids and alkaloids may be responsible for the antimicrobial action, according to Srivastava et al. [43]. In the current study, GC–MS analysis of *A. platensis* methanolic extract detected that heptadecane was the major compound found in *A. platensis* extract; this agrees with the majority of studies [44,45]. 

Heptadecane detected by GC–MS is one of the most powerful compounds with antifungal, antibacterial, antiseptic, antioxidant and germicidal properties due to its toxic potential [46]. Antimicrobial activity was demonstrated by several volatile chemicals derived from *A. platensis* preparations [26]. However, further research is needed to envision cyanobacterial crude extracts as a cheap, natural and secure supply for the pharmaceutical business following comprehensive clinical testing.

## 4. Materials and Methods

### 4.1. Cultivation of the Cyanobacteria Arthrospira platensis

*Arthrospira platensis* (axenic cyanobacterial culture) was obtained from Phycology Laboratory, Botany Department, Faculty of Science, Tanta University, Egypt. *A. platensis* was cultured [47] in Zarrouk medium; 100 mL of stock inoculant was grown in 2 L Erlenmeyer flasks with sterilized media for working culture. Cultures were incubated at 30 °C with a light intensity surface of 45 mole photon m^−2^ s^−1^, a mixture of 3% CO_2_ and 97% dry air to speed up development. 

First, bacterial air filters with a 0.45 pore diameter were used to sterilize the pumped air. The biomass of *A. platensis* was collected for this investigation at the end of the exponential phase on the 12th day. Centrifugation (Centurion Scientific, Model: CR2000, Church Farm, Stoughton, Chichester PO18, UK) at 2000× *g* for 20 min was used to collect the cyanobacterial biomass. We used distilled water to wash the pellet cells three times to remove any remaining growing medium before re-suspending them in sterile distilled water [48]. Their growth rate was followed through optical density. At 750 nm, optical density (OD) was measured using a photo colorimeter. Cultures were measured for their growth every two days [49].

### 4.2. Cyanobacterial Extract Preparation 

After drying, *Arthrospira platensis* was ground into powder. Four flasks were used with 10 g of *Arthrospira* powder combined with 150 mL of one type of solvent: ethanol for flask 1, ethyl acetate for flask 2, methanol for flask 3 and acetone for flask 4. The four flasks were left at room temperature for 5 h before being sonicated for 5 min, and centrifuged at 4000 rpm for 10 min. The resulting extracts were then resuspended in the appropriate solvent to produce a solution with a known concentration of 100 mg/mL. After this, the solvent was evaporated under low pressure to remove it. The extracts were kept at −20 °C in an airtight glass bottle for the antifungal test [50].

### 4.3. Antifungal Activity of Cyanobacterial Extracts

The agar well diffusion assay was used, as indicated by Magaldi et al. [51], to determine the antifungal activity of algal extracts against various human pathogenic fungi and to calculate the width of the inhibition zone. The tested pathogenic fungus (*Candida albicans*) was obtained from the outpatient clinic of the hospital’s dermatology department at Tanta University. *Trichophyton rubrum* AUMC No. 1804 and *Malassezia furfur* AUMC No.11710 were obtained from Moubasher Mycological Centre, Assiut University (AUMMC). Each pathogenic fungus that was examined was suspended in 0.5 mL of sterile Sabouraud’s Dextrose Agar Medium at a concentration of about 10^6^ cells per mL for *C. albicans* and *T. rubrum* and modified Dixon’s agar media for *Malassezia furfur*; they were allowed to cool before solidifying. Using a sterile cork borer, regular wells measuring 7 mm in diameter were drilled into the infected agar plates. Each well was then aseptically filled with 100 µL of each extract at a concentration of 5% that had been created using various solvents. Negative control included the use of 70% of each solvent. Positive controls included the use of clotrimazole. Plates of *T. rubrum*, *M. furfur* and *C. albicans* were incubated at 37 °C for 15 days, 28 °C for 7 days and 37 °C for 3 days, respectively, after being kept at 4 °C for 2 h to enable diffusion. The inhibition zone was measured after incubation time in triplicate.

#### Minimal Inhibitory Concentration (MIC) Assay

Cyanobacterial extracts were serially diluted to concentrations of 66, 33, 16, 8 and 4%. Wells were punched in agar media after fungal inoculation with a 10^6^ cell/mL suspension. The inoculation of wells was completed with 100 μL of diluted extracts except for the one considered as negative control which was inoculated with 100 μL of the solvent used in extraction. The MIC value was the least amount of extract necessary to prevent the growth of pathogenic fungi [52].

### 4.4. Treated Tested Fungi under Transmission Electron Microscopy (TEM)

For the TEM examination, specimens were collected by centrifugation and treated with 2.5% glutaraldehyde in a 0.1 M phosphate buffer (pH 7.4) at 4 °C for 2 h. Subsequently, 1% osmium tetroxide was applied as a post fixative (4 °C, 1.5 h). 

The sample was then dehydrated by successive dilutions of ethanol (50, 70, 90, 95 and four times 100%, each for 30 min) then dehydrated by acetone for 30 min. At the end, epoxy glue was used to implant the fixed specimens (Epoxy Embedding Medium Kit; Sigma- Aldrich, St. Louis, MO, USA). The ultramicrotome was used to cut ultra-thin and semi-thin slices (RMC PT-XL PowerTome Ultramicrotome, Wetzlar, Germany). Semi-thin (1 μm–850 nm) slices were stained with 1% toluidine blue and viewed under a light microscope using an Olympus BX61. Ultra-thin slices were cut to a thickness of 70–90 nm and stained with 2.5% uranyl acetate as the primary stain and lead citrate as the counter stain [53]. Finally, ultra-thin slices were examined using a JEM-1400 Plus (JEOL, Tokyo, Japan) transmission electron microscopy at the electron microscopy unit of the Faculty of Science at Alexandria University in Alexandria, Egypt.

### 4.5. Molecular Identification of Tested Fungus (Candida albicans)

#### 4.5.1. DNA Extraction 

The extraction of DNA was completed using the technique of Moubasher et al. [54].

#### 4.5.2. PCR and ITS Sequencing

The internal transcribed spacer (ITS) region was amplified using the universal primers ITS1 and ITS4 [55]. The same primers and (ITS1 and ITS4) are used [56].

#### 4.5.3. Phylogenetic and Alignment Analysis

Sequences of the closest and most closely related *Candida albicans* species, as well as sequences of the available type specimens, were obtained from GenBank. In this study, ITS sequences of *C. albicans* were designated as MH534924.1 in GenBank. 

*Candida albicans* sequences from the current investigation and those from GenBank were aligned using MAFFT (version 6.861b) in the default settings. BMGE processed alignment gaps and uninformative parsimony characters [57]. 

PhyML 3.0 was used to conduct maximum parsimony (MP) and maximum-likelihood (ML) phylogenetic analyses [58]. One hundred bootstrap replications were used to assess the resilience of the most sparsely branched trees [59]. The TML studies’ best optimum nucleotide substitution model was chosen using Smart Model Selection (SMS) version 1.8.1 (Moubasher Mycological Centre, Assuit, Egypt) [60]. 

### 4.6. In Vivo Study

The effect of *Arthrospira platensis* extract on the treatment of skin wounded mice artificially infected with *Candida albicans* was studied.

#### 4.6.1. Artificial Infection of Mice by Tested *Candida albicans*

Mice were prepared for infection according to Dash et al. [61]. To detect the lethal doses of *C. albicans* in the tested mice, each mouse was anesthetized by smelling ether; hair on the skin of their backs was shaved using a sterilized clipper. The cut region was sterilized with 70% ethanol. A circular lesion around 20 mm in diameter was made on the disinfected skin. Mice were injected subcutaneously with 1 mL of fungi suspension (10^6^ cfu/mL) into the wounded area [62]. 

#### 4.6.2. Preparation of *Arthrospira platensis* Extract Cream

Topical creams were made using the *A. platensis* methanolic extract in accordance with Purushothamrao et al. [63] in the Pharmaceutical Technology Laboratory, Faculty of Pharmacy, of Mansoura University. Vaseline, liquid paraffin, cetostearyl alcohol and 35 mL of deionized water composed the oil phase of the cream base, which had two phases. The aqueous phase comprised 425 mL of sodium dodecyl sulphate (SDS). 

The oil phase ingredients were melted in a China dish while being constantly stirred. The aqueous phase components were combined and warmed to temperatures close to those of the oil phase components. The aqueous phase was added in drops to the oil phase while constantly stirring until solidification. 

After cooling, we added the preservatives propyl paraben and methyl paraben. These three prepared creams were tested for topical treatment of infected mice. Nystatin was used as an antifungal agent. 

#### 4.6.3. Measurement of Wound Healing 

The cured area of the wound was measured in mm every 3 days. The following equation was then used to calculate the percentage of wound healing:

Wound healing percentage = cured area (mm) 100/20

#### 4.6.4. Treatment of Wounds Infected with the Yeast *Candida albicans* Using *Arthrospira platensis* Methanolic Extract Cream

To test the efficacy of *A. platensis* methanolic extract as a topical treatment (Table 6) for artificial skin infections, four groups of three mice were infected with the fungus on the center of the mouse back area, which was sterile before swabbing with a fresh *Candida albicans* isolate mixed with 0.1 mL of olive oil [64]. The infected area was covered with plastic film, and the mice were left for a period of time to allow the infection to form a visible lesion, which occurred after one to three days. To ensure that the infection occurred, some scales were taken from the lesion; they infected and were cultured on Sabrouaud’s dextrose agar medium with olive oil, which resulted in the growth of new *Candida albicans* colonies. 

Group 1 (G1) was left healthy as negative control. Group 2 (G2) was treated with a placebo cream base. Group 3 (G3) were treated by applying nystatin cream on the infected area and Group 4 (G4) were topically treated by applying a slim layer of methanolic extract of *A. platensis* cream on the infected wound. The diameter of each treated mouse’s wound area was measured every three days.

#### 4.6.5. Histopathological Examination of Mice Skin Tissues after Fungal Skin Infections

Histopathological examinations were performed on four mice, one from each group: the first represented healthy skin tissue as a positive control; the second represented fungi-infected skin tissue with *Candida albicans* (untreated as negative control); the third mouse showed the treated skin tissue with *A. platensis* methanolic extract cream; and the last one represented mouse skin treated with nystatin cream. This was done to differentiate between the presence and absence of infection in mouse skin tissues, as well as to demonstrate the efficacy and safety of *A. platensis* methanolic extract cream applied topically to the skin.

##### Separation of Mice Skin Tissues

Under aseptic settings, treated and control skin tissues were removed from the skin using a sterile, sharp, short-cut surgical Aicon blade. A 10% formaldehyde fixative solution was applied immediately to the excised skin sample and left to soak for 24 h.

##### Skin Sections Preparation

During a 5 min wash with distilled water to remove any remaining fixative components, the skin sample was serially dehydrated by soaking in ethanol at different ethanol concentrations (30, 50, 70 and 80%, respectively).

##### Preparation of Paraffin Blocks with Skin Sample

Skin samples were cleared for 5 min in xylene after being soaked in 95% ethanol for 5 min with traces of eosin dye to help identify them during the subsequent creation of paraffin wax (miscible with paraffin). Skin samples were placed in a molten, soft paraffin bath and kept there for 2 h to allow the paraffin to properly penetrate the skin tissues. The paraffin blocks were then solidified by immediately submerging them in cold water. By using a rotatory microtome and a clean sharp heated biconcave knife, 5 mm thick sections were cut from paraffin blocks containing skin samples (Alcon-couvreur, Rijksweg, Puurs, Belgium). Sections were distributed with a clean spatula on the slides after being floated on spotless microscopic glass slides with 2% albumin fixative heated on water vapors at 400 °C. A gelatin-blood serum mixture was used to glue the desired parts to slides. Paraffin was totally dissolved by air dryer; toluene was then adhered by hot air drier, followed by 2 min of soaking in 100% ethanol.

##### Skin Section Staining

Hematoxylin was used as the primary dye; the hematoxylin solution was composed of 2 g hematoxylin +100 mL methanol +100 mL glycerol +3 g ammonia alum +100 mL distilled water +0.24 g Na-iodate. The counter dye was eosin; the eosin solution was composed of 1 g eosin (YC. 1.45360), 5 mg glacial acetic acid and 1000 mL of 70% ethanol. Hematoxylin solution was used to stain deparaffinized slides for 20 min. Subsequently, they were rinsed in distilled water that contained 0.5% HCl in 70% ethanol for 5 min. Ammonia was added drop by drop until the nuclei were black against the white background. The slides were dehydrated in 70% ethanol. Dehydrated slides were counter stained with an eosin solution for 5 min.

The excess stain was taken out. To dehydrate the slides, ethanol dilutions were applied repeatedly. The slice was cleaned with xylene until red tissues with brown nuclei could be seen. After soaking stained sections in aqueous Hoyer mounting solution (30 g Arabic gum +200 g Chloral hydrate +16 mL glycerol +50 mL distilled water), they were permanently mounted. A glass cover was affixed to the surface and left overnight. 

#### 4.6.6. Histological Section Examination under Light Microscope

Histological examination was done in the Histology Department, Faculty of Pharmacy in Mansoura University by fixing mice lung tissues in 10% formalin solution. They were then processed and paraffin wax was embedded. Eosin and hematoxylin were used to stain blocks of skin tissue that were sectioned at a thickness of 5 μm [65]. All skin sections were photographed under a Carl Zeiss Axiosta light microscope connected with a digital Canon camera soft program zoom browser at 40× mag in the central laboratory, Faculty of Pharmacy, Mansoura University. 

### 4.7. Antioxidant Activity of Arthrospira platensis Extract

#### DPPH Assay

The DPPH (2, 2-diphenyl-1-picrylhydrazyl) radical scavenging technique was used to assess the antioxidant properties of the algal extract [66]. Methanol was used to dissolve the samples, and methanolic DPPH was used as a control. A spectrophotometer was used to measure the absorbance of the reaction mixtures at 517 nm. Ascorbic acid was utilized as a control, 

The percentage of DPPH–decolonization was determined as follows: free radical scavenging% = (Ac − As)/Ac × 100, where Ac is the absorbance of the control and As is the absorbance of the sample.

### 4.8. Cytotoxicity Assay of Arthrospira platensis Extract

Human lung fibroblast (WI38), hepatocellular carcinoma (HEPG-2), mammary gland breast cancer (MCF-7) and epithelioid carcinoma of the cervix (Hela) cell lines were obtained from Holding Company for Biological Products and Vaccines (VACSERA), Cairo, Egypt.

#### MTT Assay 

The MTT test was used on the cell lines listed above to evaluate the inhibitory effects of substances on cell proliferation [67]. Cell lines were incubated in RPMI-1640 media containing 10% fetal bovine serum. At 37 °C in a 5% CO_2_ Incubator, antibiotics of 100 units/mL penicillin and 100g/mL streptomycin were introduced. The cell lines were seeded in a 96-well plate at a density of 1.0 × 10^4^ cells/well and incubated at 37 °C for 48 h with 5% CO_2_. Following incubation, the cells were treated with various concentrations of chemicals and incubated for 24 h. After 24 h of medication treatment, 20 mL of a 5 mg/mL MTT solution was added and incubated for 4 h. To dissolve the purple formazan produced, 100 mL of dimethyl sulfoxide (DMSO) was poured into each well. A plate reader was used to measure and record the colorimetric test at 570 nm absorbance (EXL 800, Bio-Tek, Winooski, VT, USA). This was calculated as (A570 of treated samples/A570 of untreated sample) × 100.

The proportion of cell viability was estimated as (A570 of treated samples/A570 of untreated samples) × 100.

*IC_50_ (μg/mL): above 100 (non-cytotoxic), 51–100 (weak), 21–50 (moderate), 11–20 (strong), and 1–10 (very strong).

*DOX (μM): Doxorubicin as standard.

### 4.9. Gas Chromatography-Mass Spectrometry (GC-MS) Analysis of Arthrospira platensis Extract

The algal extracts were analyzed using a GC-MS Perkin Elmer Clarus 580/560 S model system (PerkinElmer, Inc. 710 Bridgeport Avenue Shelton, Connecticut 06484-4794 Waltham, MA, USA) in accordance with the customary protocol. Metabolites in extracts were identified by comparing the retention time and the fragmentation pattern with mass spectra in the NIST spectral database library software. Peak area normalization was used to express each constituent’s value of relative area (as a percentage of the total volatile composition) which was directly derived from total ion current (TIC) [68].

## 5. Statistical Methodology

All results were expressed as mean ± standard deviation. One-way analysis of variance (ANOVA test) was used for comparison among different times in the same group through quantitative data using SPSS. V. 19. (St. Louis, MO, USA) [69]. Significance was calculated at probability levels of *p* < 0.05 or *p* < 0.01.

## 6. Conclusions

In the present research, several organic extracts of *A. platensis* showed antifungal activity against *C. albicans*, *T. rubrum* and *M. furfur*. *A. platensis* methanolic extracts had the most significant antifungal effect, particularly against *C. albicans*. *A. platensis* methanolic extract showed antioxidant and cytotoxic activity. It exhibited strong cytotoxic activity against the HepG2 cell line, moderate cytotoxic activity against MCF7 and the Hela cell and weak cytotoxic activity against the WI38 cell line. In the in vivo study, the skin infected with *C. albicans* and treated with *A. platensis* methanolic extract cream had no hypha swelling and no discernible toxic consequences. The skin tissue looked normal with no inflammatory cellular infiltration. The results of GC-MS analysis demonstrated that the antifungal, antioxidant and cytotoxic properties of cyanobacterial extracts were caused by a number of bioactive components found in the *A. platensis* methanolic extract.

## Figures and Tables

**Table 1:** Antifungal activity of different *Arthrospira platensis* extracts on tested fungi.

Algal Extract	Solvents	Diameter of Inhibition Zone (mm)		
		*Candida albicans13531*	*Malassezia furfur11710*	*Trichophyton rubrum1804*
*Arthrospira platensis*	Ethanol	11.3 ± 3.11 ^c^	12 ± 2.12 ^b^	10 ± 1.11 ^a^
	Methanol	19.2 ± 3.45 ^a^	17.3 ± 2.14 ^a^	11 ± 1.12 ^a^
	Ethyl acetate	16 ± 2.17 ^b^	12.6 ± 2.15 ^b^	NA
	Acetone	16.3 ± 3.18 ^b^	10.7 ± 2.12 ^c^	NA
F-value		10.55 **	10.5 **	6.45 **
Clotrimazole		24 ± 0.01 ^a^	20 ± 0.02 ^b^	15 ± 0.01 ^c^
F-value		11.12 **		
*p* Value		0.005 **		

NA = No Activity. Each value means of three replicates ± standard deviation. For each type of algae: Means within the same column of different letters are significantly different at (*p* < 0.05). Significant at (*p* < 0.05) ** = highly significant at (*p* < 0.01). Clotrimazole 50 µg/mL was added as standard positive control for fungi.

**Table 2:** Scores of inflammation and wound healing (redness and hair growth) in mice infected with *Candida albicans* using different creams.

Scores of Inflammation and Wound Healing							
Treatments	Groups	After 4 Days		After 13 Days		After17 Days	
		Redness	Hair Growth	Redness	Hair Growth	Redness	Hair Growth
Control	G1	Non-infected					
Placebo cream	G2	++++	-	+++	-	++	-
Nystatin cream	G3	++++	-	++	+++	-	+++
*A. platensis* extract cream	G4	++	++	-	+++	-	++++

(-) Absent, (+) very Low, (++) Low, +++ Intermediate, ++++ High.

**Table 3:** DPPH radical scavenging activity (%) and IC_50_ of methanolic extract of *Arthrospira platensis*.

	Radical Scavenging %						
Conc (µg/mL)	10	20	30	40	50	60	IC_50_
Ascorbic acid	43.5 ± 0.06	49.2 ± 0.06	59.1 ± 0.1	74.2 ± 0.1	77.3 ± 0.1	79.2 ± 0.3	22 ± 0.2
*Arthrospira platensis*	25.3 ± 0.25	30.1 ± 0.26	53.4 ± 0.3	66.2 ± 0.45	75.5 ± 0.4	79.7 ± 0.21	28 ± 0.12

**Table 4:** Cytotoxicity (IC_50_) of *Arthrospira platensis* methanolic extract on different cell lines.

Compound	In Vitro Cytotoxicity IC_50_ (µg/mL)			
	WI38	HePG2	Hela	MCF7
DOX	6.72 ± 0.5	4.50 ± 0.2	5.57 ± 0.4	4.17 ± 0.2
*Arthrospira platensis*	65.82 ± 3.5	20.56 ± 1.7	38.91 ± 2.4	27.99 ± 2.1

**Table 5:** GC–MS analysis of methanolic extract of *Arthrospira platensis*.

RT	Compound Name	Norm%	MF	Biological Activity **
10.921	Ethanone, 1-(3-methylphenyl)-	0.89	C_9_H_10_O_2_	Antifungal
13.632	(Hydroxymethyl)ethylene acetate	0.58	C_7_H_12_O_5_	Antifungal, Antioxidant
13.977	Triacetin	2.34	C_9_H_14_O_6_	Antifungal, Anti-tumor, Antioxidant and Anti-inflammatory
14.377	4-Hydroxy-4-methyl-4H-naphthalen-1-one	1.46	C_11_H_10_O_2_	Antimicrobial, Antioxidant and Anti-inflammatory
16.138	Tetradecane, 2,6,10-trimethyl-	0.45	C_17_H_36_	Antioxidant, Antimicrobial
16.483	3-Buten-2-one, 4-(2,6,6-trimethyl-1-cyclohexen-1- yl)-, (E)	0.55	C_13_H_20_O	Antifungal
16.833	Butylated Hydroxytoluene	0.58	C_15_H_24_O	Antifungal, Antioxidant, Anticancer and Anti-inflammatory
17.303	(Dihydroactinidiolide) 2(4H)-Benzofuranone, 5,6,7,7a-tetrahydro-4,4,7atrimethyl-, (R)-	1.33	C_11_H_16_O_2_	Antimicrobial, Antioxidant, Anticancer and Anti-inflammatory
18.309	Hexadecane	0.89	C_16_H_34_	Antifungal, Antioxidant
19.329	11-Hexadecyn-1-ol	0.44	C_16_H_30_O	Antimicrobial
19.439	8-Heptadecene	0.78	C_17_H_34_	Antimicrobial, Antioxidant and Anti-inflammatory
19.809	Heptadecane	100.00	C_17_H_36_	Antimicrobial, Antioxidant, Anticancer and Anti-inflammatory
20.375	Tetradecane, 2,6,10-trimethyl-	0.98	C_17_H_36_	Mentioned before
21.510	3,7,11,15-Tetramethyl-2-hexadecen-1-ol	2.35	C_20_H_40_O	Antimicrobial, Antioxidant, Anticancer and Anti-inflammatory
21.600	3,7,11,15-Tetramethyl-2-hexadecen-1-ol	14.34	C_20_H_40_O	Mentioned before
21.670	1-Eicosanol	1.89	C_20_H_42_O	Antifungal, Antioxidant, Anticancer and Anti-inflammatory
21.835	3,7,11,15-Tetramethyl-2-hexadecen-1-ol	0.40	C_20_H_40_O	Mentioned before
21.910	3,7,11,15-Tetramethyl-2-hexadecen-1-ol	2.74	C_20_H_40_O	Mentioned before
22.070	3,7,11,15-Tetramethyl-2-hexadecen-1-ol	0.49	C_20_H_40_O	Mentioned before
22.150	3,7,11,15-Tetramethyl-2-hexadecen-1-ol	4.93	C_20_H_40_O	Mentioned before
22.726	(Methyl palmitate) Hexadecanoic acid, methyl ester	1.54	C_17_H_34_O_2_	Antifungal, Antioxidant, Anticancer and Anti-inflammatory
24.956	3,7,11,15-Tetramethyl-2-hexadecen-1-ol	0.68	C_20_H_40_O	Mentioned before

RT: Retention Time; MF: Molecular Formula. ** (Source: Dr. Duke’s Phytochemical and Ethnobotanical Databases).

**Table 6:** Effect of different treatments on *Candida albicans* skin infections in mice.

Mice Groups	Days	Treatment
G1	1st	Injected with 1 mL saline
	2nd–17th	no topical application
G2	1st	Injected with 10^6^ CFU/mL of tested yeast
	2nd–17th	Topical application of placebo cream base (twice daily)
G3	1st	Injected with 10^6^ CFU/mL of tested yeast
	2nd–17th	Topical application of nystatin cream (twice daily)
G4	1st	Injected with 10^6^ CFU/mL of tested yeast
	2nd–17th	Topical application of *A. platensis* methanolic extract cream (twice daily)

## Data Availability

Not applicable.
